# Impact of an Electronic Cigarette on Smoking Reduction and Cessation in Schizophrenic Smokers: A Prospective 12-Month Pilot Study

**DOI:** 10.3390/ijerph10020446

**Published:** 2013-01-28

**Authors:** Pasquale Caponnetto, Roberta Auditore, Cristina Russo, Giorgio Carlo Cappello, Riccardo Polosa

**Affiliations:** 1 CTA-Villa Chiara Psychiatric Rehabilitation Clinic and Research, Mascalucia (Catania) 95030, Italy; E-Mails: robertaauditore@virgilio.it (R.A.); kristina_russo@yahoo.com (C.R.); 2 Smoking Prevention/Cessation Centre, A.O.U, Policlinico-V. Emanuele, University of Catania, Catania 95100, Italy; E-Mail: polosa@unict.it; 3 Institute of Internal Medicine, G. Rodolico Hospital, A.O.U, Policlinico-V. Emanuele, University of Catania, Catania 95100, Italy; 4 National Strategic Planning & Analysis Research Center, Mississippi State University, Mississippi State, MS 39762, USA; E-Mail: carlocappello@live.it

**Keywords:** smoking cessation, smoking reduction, electronic cigarette, electronic nicotine delivery device, schizophrenia

## Abstract

*Background*: Cigarette smoking is a tough addiction to break. This dependence is the most common dual diagnosis for individuals with schizophrenia. Currently three effective drugs are approved for smoking cessation: nicotine replacement therapy (NRT), varenicline and bupropion. However, some serious side effects of varenicline have been reported, including depression, suicidal thoughts, and suicide. The use of bupropion also has side effects. It should not be used by people who have epilepsy or any condition that lowers the seizure threshold, nor by people who take a specific class of drugs called monoamine oxidase inhibitors. Hence, there are pharmacodynamic reason to believe they could precipitate or exacerbate psychosis. For its capacity to deliver nicotine and provide a coping mechanism for conditioned smoking cues by replacing some of the rituals associated with smoking gestures, electronic-cigarettes may reduce nicotine withdrawal symptoms without serious side effects. Our recent work with ECs in healthy smokers not intending to quit consistently show surprisingly high success rates. We hypothesised that these positive findings could be replicated in difficult patients with schizophrenia This tool may help smokers with schizophrenia remain abstinent during their quitting attempts or to reduce cigarette consumption. Efficacy and safety of these devices in long-term smoking cessation and/or smoking reduction studies have never been investigated for this special population. *Methods*: In this study we monitored possible modifications in smoking habits of 14 smokers (not intending to quit) with schizophrenia experimenting with the “Categoria” e-Cigarette with a focus on smoking reduction and smoking abstinence. Study participants were invited to attend six study visits: at baseline, week-4, week-8, week-12 week-24 and week 52. Product use, number of cigarettes smoked, carbon monoxide in exhaled breath (eCO) and positive and negative symptoms of schizophrenia levels were measured at each visit. Smoking reduction and abstinence rates were calculated. Adverse events were also reviewed. *Results*: Sustained 50% reduction in the number of cig/day at week-52 was shown in 7/14 (50%) participants; their median of 30 cig/day decreasing significantly to 15 cig/day (*p* = 0.018). Sustained smoking abstinence at week-52 was observed in 2/14 (14.3%) participants. Combined sustained 50% reduction and smoking abstinence was shown in 9/14 (64.3%) participants. Nausea was observed in 2/14 (14.4%) of participants, throat irritation in 2/14 (14.4%) of participants, headache in 2/14 (14.4%) of participants , and dry cough in 4/14 (28.6%) of participants. However, these adverse events diminished substantially by week-24. Overall, one to two cartridges/day were used throughout the study. Positive and negative symptoms of schizophrenia are not increased after smoking reduction/cessation in patients using e-cigarettes. *Conclusions*: We have shown for the first time that the use of e-cigarette substantially decreased cigarette consumption without causing significant side effects in chronic schizophrenic patients who smoke not intending to quit. This was achieved without negative impacts on the symptoms of schizophrenia as assessed by SAPS and SANS symptoms scales.

## 1. Introduction

Schizophrenia is a mental disorder characterized by a breakdown of thought processes and by poor emotional responsiveness. It is well established in studies across several countries that tobacco smoking is more prevalent among schizophrenic patients than the general population [1]. For example, in the US, 80% or more of schizophrenics smoke, compared to approximately 20% of the general population [2]. Many social, psychologic and biologic explanations have been proposed, but today research focuses on neurobiological action of nicotine and its pharmacodynamic interactions. For example, it was hypothesized that schizophrenic patients smoke to reduce symptoms and/or to mitigate the negative effects of neuroleptic therapy [3], that smoking may contribute to development of the disorder by altering neuro-chemical systems in the brain, [4] and that both conditions could arise from a common genetic vulnerability [1]. Smoking is often accepted as a customary social activity in many psychiatric treatment facilities, sometimes despite smoking bans and schizophrenic patients are seldom encouraged to quit smoking [5]. As a consequence, smoking related morbidity and mortality are particularly high in patients with schizophrenia [6].

As the risk of serious disease diminishes rapidly after quitting and life-long abstinence is known to reduce the risk of lung cancer, heart disease, strokes, chronic lung disease and other cancers [7,8], smoking cessation in these patients is mandatory.

**Figure 1:**
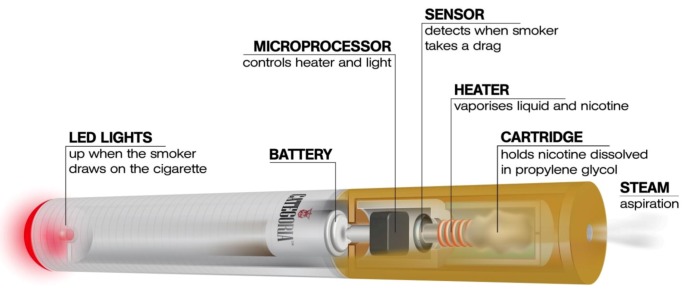
The e-cigarette is a battery-powered electronic nicotine delivery device (ENDD) designed for the purpose of providing inhaled doses of nicotine by way of a vaporized solution to the respiratory system.

Although there is little doubt that currently-marketed smoking cessation products increase the chance of committed smokers to stop smoking [9], they are not particularly effective in schizophrenic patients who smoke [5,10,11]. This scenario is further complicated by the belief that quitting smoking will worsen psychiatric symptoms, or that these patients have little or no interest in quitting. Moreover, the prescribing information for bupropion and varenicline, two important first-line medications for nicotine dependence, carry a “black-box” warning highlighting an increased risk of psychiatric symptoms and suicidal ideation in patients reporting any history of psychiatric illness [12]. A more effective approach to smoking cessation interventions in schizophrenic patients who smoke thus is an important unmet need. The electronic-cigarette (Figure 1) is a battery-powered electronic nicotine delivery device (ENDD) resembling a cigarette designed for the purpose of nicotine delivery, where no tobacco or combustion is necessary for its operation [13]. Consequently, this product may be considered as a lower risk substitute for factory-made cigarettes. In addition, people report buying them to help quit smoking, to reduce cigarette consumption and to relieve tobacco withdrawal symptoms due to workplace smoking restrictions [14]. Besides delivering nicotine, e-cigarettes may also provide a coping mechanism for conditioned smoking cues by replacing some of the rituals associated with smoking gestures. For this reason, e-cigarettes may help smokers to remain abstinent during their quit attempts or to reduce cigarette consumption. A recent internet survey on the satisfaction of e-cigarette use has reported that the device helped in smoking abstinence and improved smoking-related symptoms [15], but under acute experimental conditions, two marketed electronic cigarette brands suppressed tobacco abstinence symptom ratings without leading to measurable levels of nicotine or carbon monoxide (CO) in exhaled breath [16].

Our recent work with ECs in healthy smokers not intending to quit consistently show surprisingly high success rates [17,18]. We hypothesised that these positive findings could be replicated in difficult patients with schizophrenia. With this in mind, we designed a prospective proof-of-concept study to monitor possible modifications in the smoking habits of a group of well characterized regular smokers with schizophrenia experimenting a popular brand of e-cigarette (“Categoria”, Arbi Group Srl, Italy) focusing on smoking reduction and smoking abstinence. We also measured positive and negative symptoms of schizophrenia and possible adverse events.

## 2. Experimental Section

### 2.1. Participants

Cronic schizophrenic in-patients, who smoked ≥20 factory-made cigarettes per day (cig/day) for at least the past 10 years, able to understand the assessment procedures, and to provide written informed consent were recruited from the “C.T.A, Villa Chiara-Psichiatrica Riabilitativa e Ricerca”, Mascalucia (Catania, Italy). All patients fulfilled ICD-10 [19] and DSM-IV-TR [20] criteria for schizophrenia. The diagnosis was made by a psychiatrist and a clinical psychologist, based on definitions of these diseases in ICD-10 and DSM-IV-TR, and using a structured clinical interview [21] the Structured Clinical Interview for DSM IV Axis I Disorders (SCID-I).

None of the participants reported a history of alcohol and illicit drug use. We also excluded subjects who reported recent myocardial infarction, angina pectoris, high blood pressure (BP > 140 mmHg systolic and/or 90 mmHg diastolic), diabetes mellitus, severe allergies, poorly controlled asthma or other airway diseases. The study was approved by the local institutional ethics committee and participants gave written informed consent prior to participation in the study.

### 2.2. Study Design and Baseline Measures

**Figure 2:**
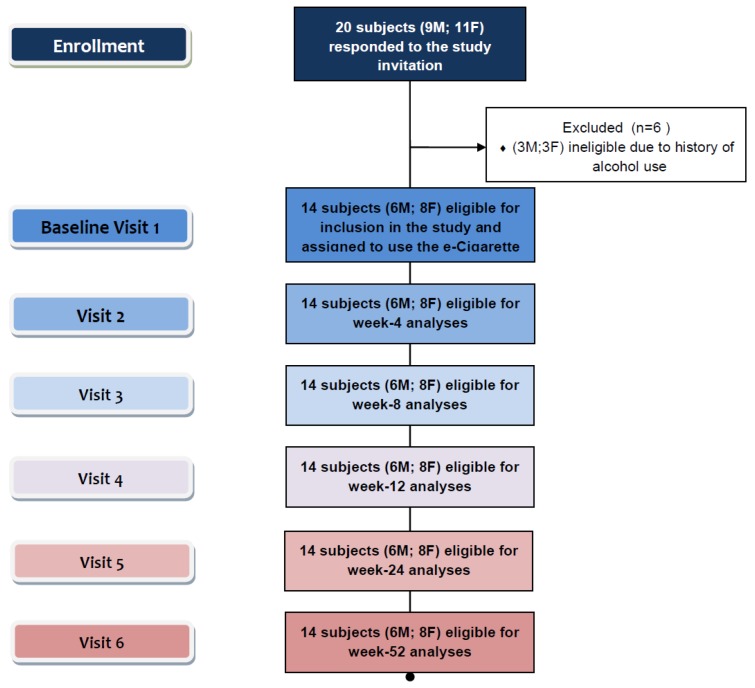
Number of patients recruited and flow of patients within the study.

Eligible participants were invited to use an electronic-cigarette (“Categoria” e-Cigarette, Arbi Group Srl, Milano, Italy) and were followed up prospectively for 12 months. They attended a total of six study visits at our smoking cessation clinic (Smoking Cessation/Research Centre, University of Catania, Italy): a baseline visit and five follow-up visits, (at week-4, week-8, week-12, week-24 and week 52) (Figure 2).

At baseline (study visit 1), basic demographic and a detailed smoking history were taken and individual pack-years (pack/yrs) calculated together with scoring of their level of nicotine dependence by means of Fagerstrom Test of Nicotine Dependence (FTND) questionnaire [22].

Positive and negative symptoms of schizophrenia were assessed with the Scale for Assessment of Negative Symptoms (SANS) [23] and the Scale for Assessment of Positive Symptoms (SAPS) [24]. Additionally, levels of carbon monoxide in exhaled breath (eCO) were measured using a portable device (Micro CO, Micro Medical Ltd, Kent, UK). Participants were given a free e-cigarette kit containing two rechargeable batteries, a charger, and two atomizers and instructed on how to charge, activate and use the e-cigarette. Key troubleshooting issues were addressed and phone numbers were supplied for both technical and medical assistance. A full 4-weeks supply of 7.4 mg nicotine cartridges (“Original” cartridges, Arbi Group Srl, Milano, Italy) was also provided and participants were trained on how to load them onto the e-cigarette’s atomizer. Random checks confirmed that the nicotine content per cartridge was 7.25 mg. Detailed toxicology and nicotine content analyses of “Original” cartridges had been carried in a laboratory certified by the Italian Institute of Health [25].

Participants were permitted to use the study product *ad libitum* throughout the day (up to a maximum of 4 cartridges per day, as recommended by the manufacturer) in the anticipation of reducing the number of cig/day smoked, and to fill out a 4-weeks’ study diary recording product use, number of any tobacco cigarettes smoked, and adverse events.

Participants were invited to came back at week-4 (study visit 2), week-8 (study visit 3), and week-12 (visit 4), (a) to receive further free supply of nicotine cartridges together with the study diaries for the residual study periods, (b) to record their eCO levels, and (c) to give back completed study diaries and unused study products.

Study participants attended two final follow-up visits at week-24 (study visit 5) and at week-52 (study visit 6) to report product use (cartridges/day) and the number of any tobacco cigarettes smoked (from which smoking reduction and smoking abstinence could be calculated), to re-check eCO levels and to assess positive and negative symptoms of schizophrenia by the Scale for Assessment of Negative Symptoms (SANS) and the Scale for Assessment of Positive Symptoms (SAPS). The SAPS consists of 31 items tapping four symptom domains and a global rating of positive symptoms (range 01–55). Higher scores indicate more positive symptoms. SAPS items are rated on a 6-point scale (0 = none, 5 = severe). The SANS is composed of 19 items measuring five domains as well as the global ratings of negative symptoms (range 0–5). Higher scores indicate more negative symptoms. SANS items are rated on a 6-point scale (0 = none, 5 = severe). Adverse events were obtained from their study diaries.

Given the observational nature of this study, no emphasis on encouragement, motivation and reward for the smoking cessation effort were provided since this study was intended to monitor the case of a smoker with schizophrenia (unwilling to quit) trying out an unconventional nicotine delivery device in a real life setting.

### 2.3. Study Outcome Measures

The primary efficacy measure was sustained 50% reduction in the number of cig/day at week-52 from baseline (*reducers*) [26]; defined as sustained self-reported 50% reduction in the number of cig/day compared to baseline for the 30 days period prior to week-52 study visit (eCO levels were measured to objectively verify smoking status and to document a reduction compared to baseline).

An additional secondary efficacy measure of the study was sustained smoking abstinence at week-52 (*quitters*); defined as complete self-reported abstinence from tobacco smoking (not even a puff) for the 30 days period prior to week-52 study visit (eCO levels were measured to objectively verify smoking status with an eCO concentration of ≤10 ppm). Those smokers who failed to meet the above criteria at the final week-52 follow-up visit (study visit 6) were categorized as reduction/cessation failures (*failures*).

### 2.4. Statistical Analyses

This was an exploratory study with opportunistic sampling and sample size calculations were not performed. Primary and secondary outcome measures were computed by including all enrolled participants. The changes from baseline (study visit 1) in number of cig/day and in eCO levels were compared with data recorded at subsequent follow-up visits using Wilcoxon Signed rank test as these methods were non-parametric. Parametric and non-parametric models were expressed as mean (±SD) and median [interquartile range (IQR)], respectively. Correlations were calculated using Spearman’s Rho Correlation. Statistical tests were 2-tailed, and *p* values of <0.05 were considered significant. The analyses were carried out using Statistical Package for Social Sciences (SPSS Inc., Chicago, IL, USA) for Windows version 17.0 [27].

## 3. Results and Discussion

### 3.1. Participant Characteristics

A total of 14 (M 6; F 8; mean (±SD) age of 44.6 (±12.5) years) chronic schizophrenic inpatients who smoked [mean (±SD) pack/years of 28.8 (±12.9)] consented to participate and were included in the study (Table 1). All fourteen patients completed the study.

**Table 1:** Patient demographics.

	Parameter	Mean (±SD) *
Subjects eligible for inclusion (n = 14)	Age	44.6 (±12.5)
	Sex	6M; 8F
	Pack Years	28.8 (±12.9)
	FTND	7 (5, 10) *
	SAPS	15 (9.5, 22) *
	SANS	44 (26.75, 53.5) *
	Cigarettes/day	30 (20, 35) *
	eCO	29 (23.5, 35.2) *
* Non-parametric data expressed as median (IQR). Abbreviations: SD: Standard Deviation; M: Male; F: Female; FTND: Fagerstrom Test of Nicotine Dependence; eCO: exhaled carbon monoxide; IQR: interquartile range; SAPS: Scale for Assessment of Positive Symptoms; SANS: Scale for Assessment of Negative Symptoms.		

### 3.2. Outcome Measures

**Figure 3:**
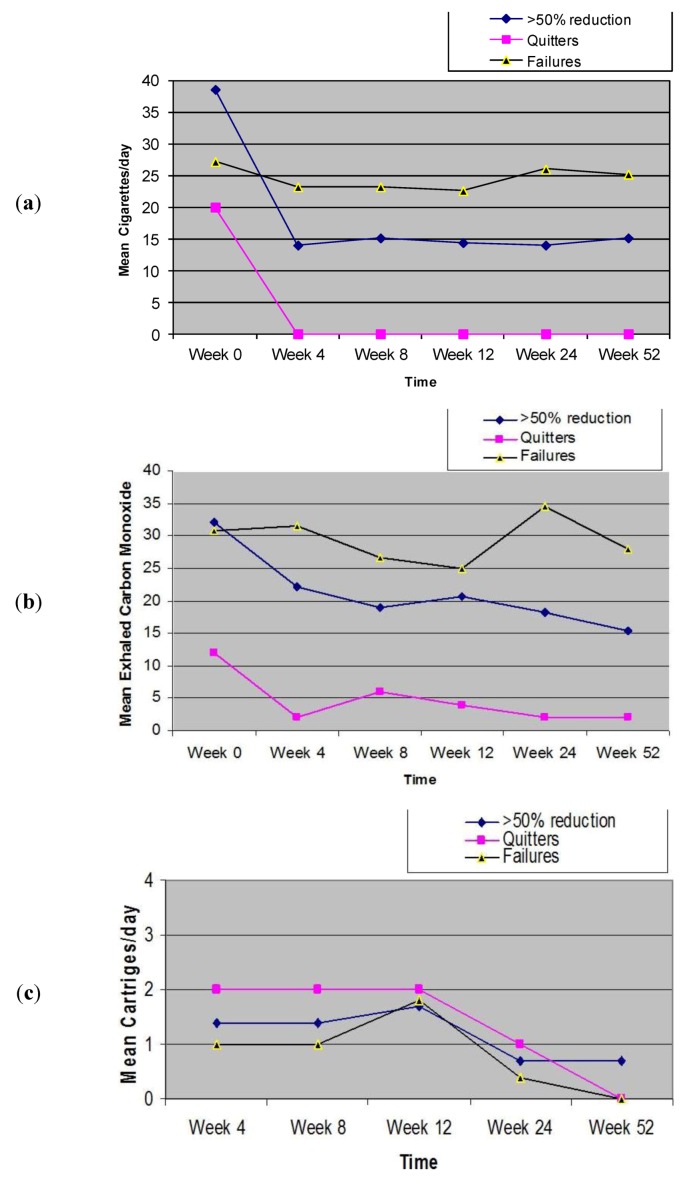
Changes in the mean (±SD) cigarette, eCO levels and cartridge use throughout the study.

Participants’ smoking status and positive and negative symptoms of schizophrenia at baseline and at 52-week is shown on Table 2. Sustained 50% reduction in the number of cig/day at week-52 was shown in 7/14 (50%) participants, with a median of 30 cig/day (IQR 30, 60) decreasing significantly to 15 cig/day (IQR 10, 20) (*p* = 0.018). There were 2/14 (14.3%) quitters. Overall, combined sustained 50% reduction and smoking abstinence was shown in 9/14 (64.3%) participants, with a median of 30 cig/day (IQR 25, 45) decreasing significantly to 12 cig/day (IQR 4.5, 17.5) (*p* = 0.007). Details of mean cigarette use are shown in Figure 3a. In the present study, the smoking reduction with “Categoria” e-Cigarette use was associated to a substantial decrease in the level of eCO (Figure 3b).

### 3.3. Product Use

Details of mean cartridge use throughout the study is shown in Figure 3c. The reported number of cartridges/day used by our study participants was dissimilar, ranging from a maximum of four cartridges/day (as per manufacturer’s recommendation) to a minimum of 0 cartridges/day (“zero” was recorded in the study diary, when the same cartridge was used for more than 24 hours). For the whole group (n = 14), a mean (±SD) 1.1 (±0.7) cartridges/day was used throughout the study. The number of cartridges/day used was slightly higher when these summary statistics were computed with the exclusion of the six study failures; the value increasing to a mean (±SD) of 1.3 (±0.5) cartridges/day. Correlation between the number of cartridges/day and smoking reduction in those participants with sustained 50% reduction in smoking was non-significant (Rho-0.809; *p* = 0.28). The correlation between the number of cartridges/day, and combined sustained 50% reduction and smoking abstinence was non-significant (Rho-0.322; *p* = 0.398).

**Table 2:** Subject parameter outcomes and psychopathological trends following 52 weeks of electronic cigarette use.

Parameter	AT BASELINE	AT 52-Weeks	*p value ^‡^*
		Post E-Cigarette	
**Sustained 50% (excluding quitters) reduction in cigarette smoking (n = 7)**			
Age	42.4 (±8.3) **^†^**		
Sex	3M; 4F		
Pack Years	34.7 (±12.1) **^†^**		
Cigarettes/day	30 (30, 60) *	15 (10, 20) *	0.018
eCO	32 (22, 39) *	17 (11, 20) *	0.028
SAPS	15 (12, 23) *	12 (10, 25) *	0.147
SANS	51 (41, 63) *	45 (40, 48) *	0.351
**Sustained 100% (quitters) reduction in cigarette smoking (n = 2)**			
Age	51(±7.1) **^†^**		
Sex	1M; 1F		
Pack Years	20.25 (±0.0) **^†^**		
Cigarettes/day	20 (15, 15) *	0 (0, 0) *	0.157
eCO	24 (15.7, 20. 3) *	2 (1.5, 1.5) *	0.180
SAPS	13 (3, 16.5) *	14 (4.5, 16.5) *	0.317
SANS	27.5 (7.5, 33.8) *	26.5 (7.5, 32.2) *	0.317
**Sustained >50% (including quitters) reduction in cigarette smoking (n = 9)**			
Age	44.3 (±8.5) **^†^**		
Sex	4M; 5F		
Pack Years	31.5 (±12.2) **^†^**		
Cigarettes/day	30 (25, 45) *	12 (4.5, 17.5) *	0.007
eCO	22 (15, 32) *	12 (6, 15.5) *	0.021
SAPS	15 (10, 22.5) *	12 (10, 22.5) *	0.203
SANS	48 (35.5, 62) *	45 (39, 27.5) *	0.260
**Smoking Failure (<50% smoking reduction) (n = 5)**			
Age	40.6 (±17.7) **^†^**		
Sex	2M; 3F		
Pack Years	23.9 (±14.3) **^†^**		
Cigarettes/day	21(17.5, 40) *	21 (17.5, 35) *	0.317
eCO	28 (25, 38) *	29 (20, 35.5) *	0.345
SAPS	12 (9, 18.5) *	11 (9, 17) *	0.581
SANS	30 (13.5, 48.5) *	32 (14.5, 45) *	0.684

Abbreviations: SD: Standard Deviation; M: Male; F: Female; eCO: exhaled carbon monoxide. **^‡^*** p* value: within group Wilcoxon Signed Rank Test. **^†^** Parametric data expressed as mean (±SD). * Non-parametric data expressed as median [interquartile range (IQR)].

### 3.4. Adverse Events

The most frequently reported adverse events were nausea, shown in 2/14 (14.4%), throat irritation shown in 2/14 (14.4%), headache shown in 2/14 (14.4%), and dry cough shown in 4/14 (28.6%) (Table 3). Table 4 shows the distribution of the four most commonly reported adverse events (AEs), separately for failures, reducers, abstainers. These events were most commonly reported at the beginning of the study and appeared to wane spontaneously by study visit 5. Withdrawal symptoms were absent (*i.e.*, depression, anxiety, insomnia, irritability, hunger, constipation were not reported). Moreover, no serious adverse events (*i.e.*, events requiring unscheduled visit to the family practitioner or hospitalization) occurred during the study.

**Table 3:** Adverse events reported by participants who completed all study visits.

Adverse Event	Study Visits				
	4-week n/n (%)	8-week n/n (%)	12-week n/n (%)	24-week n/n (%)	52-week n/n (%)
Throat irritation *	1/14 (7.2%)	2/14 (14.4%)	0/14 (0%)	0/14 (0%)	0/14 (0%)
Mouth Irritation *	0/14 (0%)	0/14 (0)	0/14 (0%)	0/14 (0%)	0/14 (0%)
Sore Throat	0/14 (0%)	0/14 (0)	0/14 (0%)	0/14 (0%)	0/14 (0%)
Dry cough	4/14 (28.6%)	4/14 (28.6%)	1/14 (7.2%)	0/14 (0%)	0/14 (0%)
Dry mouth	0/14 (0%)	0/14 (0)	0/14 (0%)	0/14 (0%)	0/14 (0%)
Mouth ulcers	0/14 (0%)	0/14 (2.9%)	0/14 (0%)	0/14 (0%)	0/14 (0%)
Dizziness ^§^	0/14 (0%)	0/14 (0)	0/14 (10%)	0/14 (0%)	0/14 (0%)
Headache	2/14 (14.4%)	1/14 (7.2%)	1/14 (7.2%)	0/14 (0%)	0/14 (0%)
Nausea	2/14 (14.4%)	0/14 (0%)	1/14 (7.2%)	0/14 (0%)	0/14 (0%)
Depression	0/14 (0%)	0/14 (0%)	0/14 (0%)	0/14 (0%)	0/14 (0%)
Anxiety	0/14 (0%)	0/14 (0%)	0/14 (0%)	0/14 (0%)	0/14 (0%)
Insomnia	0/14 (0%)	0/14 (0%)	0/14 (0%)	0/14 (0%)	0/14 (0%)
Irritability	0/14 (0%)	0/14 (0%)	0/14 (0%)	0/14 (0%)	0/14 (0%)
Hunger	0/14 (0%)	0/14 (0%)	0/14 (0%)	0/14 (0%)	0/14 (0%)
Constipation	0/14 (0%)	0/14 (0%)	0/14 (0%)	0/14 (0%)	0/14 (0%)

* Throat and mouth irritation were described either as tickling, itching, or burning sensation. **^§^** Dizziness, was also used to mean vertigo and light-headedness.

**Table 4:** Distribution of the four most commonly reported adverse events (AEs), separately for failures, reducers, abstainers.

AEs	4-week	8-week	12-week	24-week	52-week
Dry cough	failures (n 2)	failures (n 2)	failures (n 0)	failures (n 0)	failures (n 0)
	reducers (n 1)	reducers (n 1)	reducers (n 1)	reducers (n 0)	reducers (n 0)
	abstainers (n 1)	abstainers (n 1)	abstainers (n 0)	abstainers (n 0)	abstainers (n 0)
Headache	failures (n 0)	failures (n 0)	failures (n 0)	failures (n 0)	failures (n 0)
	reducers (n 1)	reducers (n 0)	reducers (n 0)	reducers (n 0)	reducers (n 0)
	abstainers (n 1)	abstainers (n 1)	abstainers (n 1)	abstainers (n 0)	abstainers (n 0)
Nausea	failures (n 1)	failures (n 0)	failures (n 0)	failures (n 0)	failures (n 0)
	reducers (n 0)	reducers (n 0)	reducers (n 0)	reducers (n 0)	reducers (n 0)
	abstainers (n 1)	abstainers (n 0)	abstainers (n 0)	abstainers (n 0)	abstainers (n 0)
Throat irritation	failures (n 0)	failures (n 1)	failures (n 0)	failures (n 0)	failures (n 0)
	reducers (n 1)	reducers (n 1)	reducers (n 0)	reducers (n 0)	reducers (n 0)
	abstainers (n 0)	abstainers (n 0)	abstainers (n 0)	abstainers (n 0)	abstainers (n 0)

### 3.5. Positive and Negative Symptoms of Schizophrenia

Positive and negative symptoms of schizophrenia are not increased after smoking reduction/cessation in patients using e-cigarettes (Table 2). Other studies suggests that positive and negative symptoms of schizophrenia are not increased after smoking cessation in patients receiving nicotine patches or placebo patches [28], after smoking reduction following a treatment of bupropion [29], or after smoking reduction following a treatment of nicotine patches [30].

### 3.6. Discussion

We have shown for the first time that the use of e-cigarettes substantially decreased cigarette consumption without causing significant side effects in chronic schizophrenic patients who smoke. This was achieved without negative impacts on symptoms of schizophrenia as assessed by SAPS and SANS symptoms scales.

Severity of nicotine dependence, smoking prevalence, and the likelihood of success of quit attempts are much worse in schizophrenia than in patients with other mental disorders or smokers in the general population. Therefore, our findings may be of great importance.

Smokers with schizophrenia may use nicotine as a self-medication for the illness. The self medication hypothesis is supported by study showing that smoking can transiently reverse the deficit [6] in the processing of auditory stimuli that is found in patients with schizophrenia [31] and by research suggesting that smoking cigarette has a beneficial effect on visuospatial working memory in smokers with schizophrenia [32].

Patients with schizophrenia may also smoke to offset the side effects of antipsychotic drugs, as suggested by research showing that a nicotine patch attenuates the adverse side effects of these drugs [33] and that cigarette smoking reduces neuroleptic-induced parkinsonism [34].

Another hypothesis is that some antipsychotic drugs may increase smoking, as suggested by research showing that haloperidol caused a dose-related increase in *ad lib* smoking in patients with schizophrenia, in comparison with their baseline level when they were taking no antipsychotic medications [35].

A further hypothesis is that genetic factors explain the co-occurrence of smoking and schizophrenia [36], as suggested by research showing that nicotinic receptors are abnormally expressed [37] and function abnormally in people with schizophrenia [38].

In this pilot study, we have shown for the first time that substantial and objective modifications in the smoking habits may occur in smokers with schizophrenia using e-cigarettes, with significant smoking reduction and smoking abstinence and no apparent increase in withdrawal symptoms and in positive and negative symptoms of schizophrenia. Chronic schizophrenic patients using e-cigarettes substantially decreased cigarette consumption with an overall quit rate in 2/14 (14.3%) at week-52. Moreover, at least 50% reduction in cigarette smoking was observed in 7/14 (50%) of participants. Overall, combined reduction and smoking abstinence was shown in 9/14 (64.3%) of participants. Some of the smoking/reduction failures could have been related to malfunctions and technical failures of the product tested in the present study. 

These preliminary findings are of great importance considering that chronic schizophrenic patients who smoke are generally not interested in quitting. The large magnitude of this effect suggests the e-cigarette may be a valuable tool of tobacco harm reduction in this special population. These positive findings may be explained by the great compensatory effect of e-cigarettes at both physical and behavioral level [9,10,11,12,13,14]; in particular these products are known to provide a coping mechanism for conditioned smoking cues by replacing some of the rituals associated with smoking gestures (e.g., hand-to-mouth action of smoking). In agreement with this, we have recently demonstrated that nicotine free inhalators can only improve quit rates in those smokers for whom handling and manipulation of their cigarette played an important role in their ritual of smoking [39].

The most frequent adverse events reported by our patients were throat irritation, nausea, headaches and dry cough, but all appeared to wane spontaneously with time. Throat irritation and dry cough are likely to be secondary to exposure to propylene glycol mist generated by the e-cigarette’s atomizer. Propylene glycol is a low toxicity compound widely used as a food additive and in pharmaceutical preparations. Exposure to propylene glycol mist may occur from smoke generators in discotheques, theatres, and aviation emergency training and is known to cause ocular, mouth, throat, upper airway irritation and cough [40,41].

In contrast with other ENDDs that are known to generate substantial level of eCO [42], in the present study, the smoking reduction/cessation with “Categoria” e-cigarette use was associated to a substantial decrease in the level of eCO. This is in agreement with previous studies [17,18].

Therefore, the e-cigarette can be seen as a safe harm-reduction strategies for smokers with schizophrenia. Harm-reduction strategies are aimed at reducing the adverse health effects of tobacco use in individuals unable or unwilling to quit. Substantially reducing the number of cig/day is one of several kinds of harm reduction strategies [43]. Here, we propose an alternative harm reduction approach for patients with schizophrenia with the e-cigarette being used as a safe alternative source of nicotine for patients who smoke.

It is uncertain whether substantial smoking reduction in smokers using the e-cigarette will translate in health benefits, but a number of studies have analyzed the ability of smoking reduction to lower health risks and have reported some reductions in cardiovascular risk factors and lung cancer mortality [44,45,46]. Moreover, reduction in cigarette smoking by e-cigarette may well increase motivation to quit as indicated by a substantial body of evidence showing that gradually cutting down smoking can increase subsequent smoking cessation among smokers [47,48,49,50].

There are some limitations in our study. Firstly, this was a small uncontrolled study, hence the results observed may be due to a chance finding and not to a true effect; consequently the results should be interpreted with caution. However, it would have been quite problematic to have a placebo arm in such a study. Secondly, this is not an ordinary cessation study and therefore direct comparison with other smoking cessation products cannot be made. Lastly, assessment of withdrawal symptoms in our study was not rigorous. Withdrawal was assessed at each visit by simply asking about the presence/absence of irritability, restlessness, difficulty concentrating, increased appetite/weight gain, depression or insomnia. It is likely that this way of collecting information is liable to recall bias. Therefore, the reported lack of withdrawal symptoms in the study participants should be considered with caution [4].

## 4. Conclusions

Even with intensive smoking cessation management programs specifically designed for patients with schizophrenia, quit rates are low [51]. Although not formally regulated as a pharmaceutical product, the e-cigarette can help smokers with schizophrenia to reduce their cigarette consumption or remain abstinent and reduce the burden of smoking-related morbidity and mortality, particularly in schizophrenic patients who smoke. However, large and carefully conducted RCTs, among healthy smokers and among persons with more common mental health problems like anxiety ordepression, will be required before a definite answer about the efficacy and safety of these devices can be formulated.
